# Daily Steps During Nutritional Lifestyle Modification Programs for Obesity Management: A Systematic Review and Meta-Analysis

**DOI:** 10.3390/ijerph23040522

**Published:** 2026-04-17

**Authors:** Dana Saadeddine, Matteo Foglia, Elisa Berri, Silvia Raggi, Leila Itani, Marwan El Ghoch

**Affiliations:** 1 Center for the Study of Metabolism, Body Composition and Lifestyle, Department of Biomedical, Metabolic and Neural Sciences, University of Modena and Reggio Emilia, 41125 Modena, Italy; dana.saadeddine@unimore.it; 2 Degree Course of Dietetics, Innovation and Research Training Service, Azienda Ospedaliero-Universitaria di Modena, 41124 Modena, Italy; 339720@studenti.unimore.it (M.F.); elisa.berri@unimore.it (E.B.); silvia.raggi@unimore.it (S.R.); 3 Department of Primary Care, Azienda Unità Sanitaria Locale-IRCCS di Reggio Emilia, 42123 Reggio Emilia, Italy; 4 Department of Nutrition and Dietetics, Faculty of Health Sciences, Beirut Arab University, P.O. Box 11-5020 Riad El Solh, Beirut 1107 2809, Lebanon; l.itani@bau.edu.lb

**Keywords:** pedometer, BMI, overweight, weight loss, weight maintenance, meta-regression

## Abstract

**Highlights:**

**Public health relevance—How does this work relate to a public health issue?**

- Obesity is a growing public health issue, as by 2035 its prevalence is forecast to exceed 30% of the global population.
- The identification of novel strategies that improve obesity treatment outcomes is a priority for public health.

**Public health significance—Why is this work of significance to public health?**

- On a general level, the significance of this work to public health lies in assessing the association between lifestyle modification programs and weight-related outcomes, given their potential as an affordable way for obesity management.
- Specifically, the findings suggest that higher daily step counts may be associated with improved outcomes in obesity treatment, highlighting a simple and feasible behavior that could be considered within lifestyle interventions.

**Public health implications—What are the key implications or messages for practitioners, policy makers and/or researchers in public health?**

- Policy makers should consider lifestyle modification as a valid treatment for obesity, since it determines a significant weight loss in the long term, and as such, should be considered a first-attempt intervention.
- During lifestyle modification programs for obesity, practitioners may encourage patients to increase their average daily steps, as this appears to be associated with better clinical outcome.

**Abstract:**

Background and objectives: Increasing daily steps during weight management programs remains one of the most common recommendations; however, why, when and how many is still unclear. To clarify this, we aim to conduct a systematic review and meta-analysis. Methods: The study was conducted in adherence to the PRISMA guidelines on randomized controlled trials (RCTs), that included weight loss (WL) interventions based on lifestyle modification programs (LSMs), compared to “as usual care” considered as controls, to whom both daily steps and WL% were reported or retrievable at baseline (Time 0), end of WL phase (Time 1, WL1%), and weight maintenance phase (Time 2, WL2%), for both arms. Results: A total of 18 RCTs met the inclusion criteria and were included in the systematic review. Of those, 14 underwent meta-analysis and five main findings were revealed: (i) at baseline (Time 0), no significant difference was observed in mean daily steps between the LSM and controls (7280 vs. 7180, *p* = 0.336), reflecting a similar lifestyle between arms; (ii) at Time 1, the mean duration was 7.88 months (range = 3–12 months), and the LSM arm showed a significant increase in daily steps with respect to baseline (8454 vs. 7486 steps, *p* = 0.017) and a significant WL (WL1% = 4.39%, *p* < 0.001); (iii) at Time 2, the mean duration was 10.27 months (range = 3–24 months), and the LSM arm maintained the level of daily steps achieved by the end of WL phase (8241 vs. 8454 steps, *p* > 0.05), and also a significant WL% (WL2% = 3.28%, *p* = 0.001); (iv) the control arm showed no significant changes in daily steps and weight status at all times of assessment; and (v) the meta-regression showed in the LSM arm a positive relationship between daily steps at Time 1 (β = 1.33, *p* = 0.03) and Time 2 (β = 1.10, *p* = 0.02), both with WL2%. Conclusions: Our preliminary study results support that during LSM programs, patients should be encouraged to increase their daily steps during the WL phase, targeting approximately 8500 steps/day and maintaining these levels during the maintenance phase, since this strategy appears to be a useful behavioral approach associated with maintaining significant WL in the long term.

## 1. Introduction

Recently, the World Health Organization (WHO) published the regional obesity report, which claims that more than 60% of the European population is affected by overweight or obesity [1]. It is predicted that this scenario will get worse in the coming decade, according to the projections of the World Obesity Atlas Report, whereby the 2035 prevalence of obesity is forecast to exceed 30% of the global population [2]. This projected rise is a considerable source of concern due to the strong association of overweight and obesity with several medical and psychological comorbidities [3,4,5], with a heavy financial burden on healthcare systems [6] as well as the social impacts that unavoidably lead to major disabilities and increased risk of mortality [7]. For this reason, international associations dealing with obesity have promoted research roadmaps [8] and clinical guidelines [9] to improve the management of overweight and obesity with a wide range of recommended interventions from nutritional therapy [10,11] to anti-obesity drugs [12] and bariatric surgery [13], as well as their use in combination to improve clinical outcomes [14,15].

Lifestyle modification (LSM) programs remain the cornerstone intervention for treating overweight and obesity [16,17]. They usually combine dietary and physical activity recommendations with behavioral strategies to determine changes that counter overeating and sedentary habits, leading to caloric restriction and deficit that determine a significant weight loss (WL) [18,19]. Usually, in weight management clinical practice, increasing daily steps remains one of the most common and suggested strategies, highlighted and underlined by obesity specialists when delivering WL interventions to their treatment-seeking patients with overweight and obesity [20,21,22]. For example, one of the most used strategies in real-world weight management settings is to encourage patients to monitor their number of daily steps using a pedometer, and then to add a small number of steps (i.e., 250–500) at 2–3-day intervals up to a target value of 10,000–12,000 steps/day. However, concerns have been raised regarding this approach in clinical practice, because, first of all, there is no robust data regarding the positive association between achieving more daily steps and having better weight management outcomes. Indeed, some studies found that the increase in daily steps in people with overweight or obesity does not determine significant changes in weight status, nor is it able to prevent weight regain in this population [23,24]. Second, supposing that this association does exists, practically it is not known during which period of the weight management program the increase in daily steps becomes influential and more importantly, questions still remain regarding when patients should start increasing their daily steps, i.e., in the early or late stages of these programs (e.g., during the WL phase or/and during the weight maintenance phase). Last but not least, there is a lack of clarity on the number of daily steps patients should be aiming for during LSM programs. Based on this situation, the current available recommendations have been established using broad ranges or, in some cases, have been determined arbitrarily (i.e., 10,000 steps/day), leaving both obesity specialists and patients with no clear and precise indications.

In light of these considerations and to address these gaps, we aim to conduct a systematic review and meta-analysis with the primary objective to (1) quantify the effect of LSM interventions on the daily step counts compared to usual care management during both WL and weight maintenance phases and (2) to examine whether the timing of changes in daily step counts across these phases, are associated with weight loss outcomes. This review is conducted in accordance with the PICO process [25], as detailed below.

P—Population: Adult participants of both genders with overweight or obesity, however defined, with or without comorbidities. I—Intervention: LSM intervention mentioned or identified as so, because it included one or more strategies based on behavioral or cognitive behavioral treatment aiming to determine WL or/and followed by a weight maintenance phase, during which an objective assessment of daily steps are reported or retrievable, at baseline (Time 0), end of WL phase (Time 1), and weight maintenance phase (Time 2). C—Comparison: Non-pharmacological or surgical management of patients with overweight or obesity seeking treatment in a clinical practice with “as usual care”, such as weight management based on a prescriptive approach (i.e., diet), or a “no treatment or/and on a waiting list”, and availability of an objective assessment of daily steps are reported or retrievable, at all times T0, T1 and T2 of follow-up. O—Outcome: WL% after the WL phase (WL1%) and after the end of the weight maintenance phase (WL2%), as well as mean daily steps at baseline, end of the weight loss phase and weight loss maintenance phase.

## 2. Methods

The systematic review was conducted according to the Preferred Reporting Items for Systematic reviews and Meta-Analyses (PRISMA) guidelines [26] and registered in the International Prospective Register of Systematic Reviews (PROSPERO) [27] as “Physical Activity Strategies and Weight Maintenance: A systematic Review of Randomized Controlled Trials and Meta-Analysis” (PROSPERO no. CRD420251150003).

### 2.1. Inclusion and Exclusion Criteria

All studies were included if they met the following criteria: (i) written in English; (ii) conducted on adults with overweight or obesity; and/or (iii) studies with a randomized controlled design. No other studies of different design, such as prospective or retrospective observational (analytical or descriptive), experimental or quasi-experimental non-controlled studies, reviews, cross-sectional studies or non-original articles (i.e., case reports, editorials, “Letters to the Editor” or book chapters) were included. No time limit on the date of publication was set.

### 2.2. Information Source and Search Strategy

The literature search was designed and performed independently in duplicate by four authors, namely, the two principal (DS and MF) and two senior investigators (ME and LI). The PubMed/Medline and Scopus databases were systematically screened using the following MeSH terms and their combinations.

PubMed/Medline database: ((((“Weight Loss”[MAJR])) OR (“Body Weights and Measures”[MeSH])) OR (Weight loss maintenance OR Maintain weight loss OR Long-term weight management OR Sustainable weight loss OR Post-diet weight control OR Prevent weight regain OR Weight loss plateau OR Weight loss journey OR Weight loss success stories OR Weight loss transformation) AND ((“wearable device*” OR “fitness tracker” OR “pedometer” OR “ARMBAND” OR “ACTIGRAPH” OR “biometric monitoring device” OR “steps” OR “step count” OR “steps per day” OR “steps/day” OR pedomet* OR acceleromet*)) AND (((“Weight Reduction Programs”[Mesh]) OR (“Weight Loss”[MAJR])) OR (“Behavior Therapy”[MeSH]))).

Scopus database: (((“Weight Loss”) OR (“Body Weights and Measures”) OR (Weight loss maintenance OR Maintain weight loss OR Long-term weight management OR Sustainable weight loss OR Post-diet weight control OR Prevent weight regain OR Weight loss plateau OR Weight loss journey OR Weight loss success stories OR Weight loss transformation)) AND ((“wearable device” OR “fitness tracker” OR “pedometer” OR “ARMBAND” OR “ACTIGRAPH” OR “biometric monitoring device”)) AND ((“Weight Reduction Programs”) OR (“Weight Loss”) OR (“Behavior Therapy”))).

Moreover, a manual search was carried out to retrieve other articles that had not been identified via the initial search strategy. Publication date was not considered an exclusion criterion for the purposes of this review.

### 2.3. Study Selection, Quality, and Risk-of-Bias Assessments

Two authors (DS and MF) independently screened the resulting articles for their methodology and appropriateness for inclusion. The quality of each study was determined according to the Jadad scale [28], which relies on three different criteria: randomization (two points), blinding (two points) and a description of dropouts (one point). Out of a total of five points, a score ≥ 3 is indicative that the trial is of good quality [28]. The Jadad scale was selected as a simple and widely used tool to assess core methodological quality domains and allow for consistent and efficient evaluation across studies; however, to address its known limitations, additional risk-of-bias criteria were applied to capture aspects not fully covered by the scale. The risk of bias tool was used to determine whether the standards of reporting for RCTs were satisfied [29] according to the following items: the randomization method, allocation sequence concealment, outcome measurement, interventionist training, withdrawals, intent-to-treat analyses, clustering and baseline characteristics. Studies were assigned a “yes” for each criterion they satisfied, and a “no” for each they did not, while “not reported” was used where information for evaluation was insufficient or unavailable. Consensus discussion between the principal (DS and MF) and senior (ME and LI) investigators was used to resolve disagreements between reviewers.

### 2.4. Data Collection Process and Data Items

The title and abstract of each paper were first assessed by two independent authors (DS and MF) for language suitability and subject matter relevance, and the studies thereby selected were assessed in terms of their appropriateness for inclusion and the quality of the method. The results of those studies passing both rounds of screening are shown in Table 1.

### 2.5. Data Extraction

Information at different time points (T0, T1 and T2) were extracted on intervention type (LSM/control), duration of intervention, age, gender (whenever reported), number of participants in each arm, mean WL% from baseline [standard error (SE)/standard deviation (SD)/95% confidence interval (CI)] (when reported) and mean daily steps per day (SE/SD/95% CI). When WL%s and their SD or SE were not reported, they were calculated by linear rescaling of absolute change scores using the reported mean weight at baseline (time T0), at the end of the WL phase (T1) and at the end of the weight maintenance phase (T2) [30]. The SD for the absolute mean change from baseline at time T1 and time T2 was then imputed using SD0 and SD1/SD2, respectively, using a correlation of 0.95 based on the reported correlation for WL [30,31]. The WL% for each time point was calculated by dividing the value of change from baseline (CFB) at each time point (T1 and T2) by the mean weight at baseline (T0) and multiplying by 100. Under linear rescaling, the variance scales proportionally with the transformation factor; hence, the SD for WL% was calculated by dividing the SD of CFB by weight at baseline and multiplying by 100 [32]. A similar approach was used to estimate total steps when only the change from baseline was reported [30]. A correlation of 0.8 was assumed based on the Cochrane guidance when the correlation is not reported [31]. This is in line with reports from other systematic reviews and other studies reflecting the stability of step counts over time with an interclass correlation (ICC) of 0.8 [33,34]. When the SE was not reported, it was calculated using the reported SD or the reported 95% CI and the reported “*n*” for each arm.

### 2.6. Meta-Analysis

The primary outcome of this study was daily step counts, reflecting the behavioral target, with weight loss percentage as a secondary clinical outcome. The analysis was structured to first present weight loss outcomes to provide a clinical context, followed by analysis of daily step counts as the primary behavioral outcome, and finally to explore the association between step counts and weight loss using meta-regression. The meta regression was conducted as an exploratory analysis within the LSM arm.

A random effects inverse variance meta-analysis was conducted to estimate the pooled mean WL% in the LSM arm(s) or control arm(s) at the end of each WL phase (T1) and weight maintenance phase (T2), as well as the pooled mean daily step counts and differences in counts at T0, T1 and T2. Use of the inverse variance meta-analysis allows for weighing the studies by their sample size. A restricted maximum likelihood random-effects meta-analysis with Hartung–Knapp correction was used to account for uncertainty in estimating between-study heterogeneity and to avoid Type I errors. Between-study heterogeneity was assessed using the I^2^ statistic [31,35]. A Wald z-test was used to compare pooled effect size within groups over time and between groups (LSM vs. control) at the same time points [36]. Only studies that had both daily step counts and WL% at each time point within each arm were included in the meta-analysis of pooled estimates. This ensured alignment of exposure and outcome within the same participants at one time point. As for the pooled mean difference (MD) in step counts, only studies that have paired step counts in intervention and control groups at the same time point were included.

### 2.7. Assessing Risk of Publication Bias

To assess the risk of publication bias or influence of small studies, funnel plots of study-level effect measure and their standard errors were examined, and Egger regression tests were used [37]—an insignificant Egger test (*p* > 0.05) indicated no evidence of asymmetry. A sensitivity analysis was undertaken for all primary analyses by comparing the pooled effect size estimate from fixed effects models and random effects models [31,35]. In the case where asymmetry was suggested (*p* < 0.05), the results from the fixed effects were examined and compared regarding direction and magnitude of effect and statistical significance [31,35]. However, funnel plot asymmetry was interpreted with caution, given that it may arise from other factors, including heterogeneity between studies arising from differences in methodological quality, clinical settings, types of participants or interventions, the study size–effect relationship, having fewer than 10 studies, and chance rather than bias alone [31,37,38].

### 2.8. Meta Regression

The meta-regression analysis was conducted as an exploratory analysis within LSM arms separately to assess the association between daily step count and WL%. In the meta-regression model, WL% was the outcome, and the study-level predictors were the daily steps at T0, T1 and T2. Daily step counts were rescaled to 1000 steps per day to facilitate interpretability. Meta-regression was conducted using a random effects model with restricted maximum likelihood estimator and Hartung–Knapp correction to account for between-study variability [35].

The analysis was done using the R (version 4.5.2) Rstudio integrated development environment (version 2025.9.2.418). The metaphor R package was used to fit the models for meta-analysis and meta-regression, produce forest plots, funnel plots, and perform the Egger test [39].

### 2.9. Weight Loss and Weight Maintenance Duration

A weighted estimate of the mean duration for the WL phase, the weight maintenance phase and the entire duration of the study was calculated as the average duration for each phase weighted by the number of participants in the studies included at T0.

### 2.10. Sensitivity Analysis

To assess the robustness of the findings, several sensitivity analyses were conducted addressing different sources of uncertainty. First, pooled estimates were compared using both fixed effects and random effects models to evaluate the impact of model specifications. Second, given that change-score variances were derived using an assumed within-study correlation coefficient (*r* = 0.95 for WL% and *r* = 0.8 for steps), additional analyses were performed using alternative plausible values (*r* = 0.6, *r* = 0.7 and *r* = 0.9). Additionally, leave-one-out analyses were done by iteratively excluding each study to evaluate the influence of individual studies on pooled estimates of heterogeneity. Influence diagnostics were also performed using standardized residuals, Cookes’s distance, difference in fits (DFFITS), covariance ratios, and leverage (hat values) to identify potentially influential or outlying studies. These analyses were conducted for all primary outcomes and time points.

## 3. Results

The initial search retrieved 868 papers, and 105 were immediately eliminated as duplicates, with 763 remaining screened reports. In the first round of screening, 50 papers were excluded on the following grounds: (i) not written in English; (ii) not conducted in humans. In the second round 713 papers were screened on the basis of title and abstract and 260 papers were excluded due to the following reasons: (i) not related to the topic; (ii) a review article (systematic review, meta-analysis, or narrative review), study design or protocol; (iii) not a journal article, no abstract available, abstract only, and/or no author found. In the third round, 453 full-text papers were assessed for eligibility. Among these, 300 were removed on the basis of: (i) not being conducted on individuals with overweight or obesity; (ii) not being related to physical activity; (iii) not dealing with a WL intervention; (iv) not reporting the body weight; and (v) not reporting the number of daily steps. In the fourth round, 133 papers were excluded on the basis of: (i) not being an RCT; (ii) not reporting the weight maintenance phase. Lastly, out of 20 articles, nine were removed because they: (i) did not distinguish between intervention and control groups and (ii) implemented physical activity as the only intervention, rather than embedded in an LSM program [40,41,42,43,44,45,46,47,48].

Moreover, the manual search identified 111 papers through websites and bibliographic references. Among these, 74 were selected for retrieval, while 37 were excluded for: (i) being systematic reviews or meta-analyses; (ii) not being an RCT. Following a full-text review, 59 papers were further excluded for not reporting a weight maintenance period. To sum up, eight out of 15 articles were eliminated due to the following reasons: (i) not distinguishing between intervention and control groups; (ii) implementing physical activity as the only intervention, rather than embedded in an LSM program [49,50,51,52,53,54,55,56].

**Figure 1:**
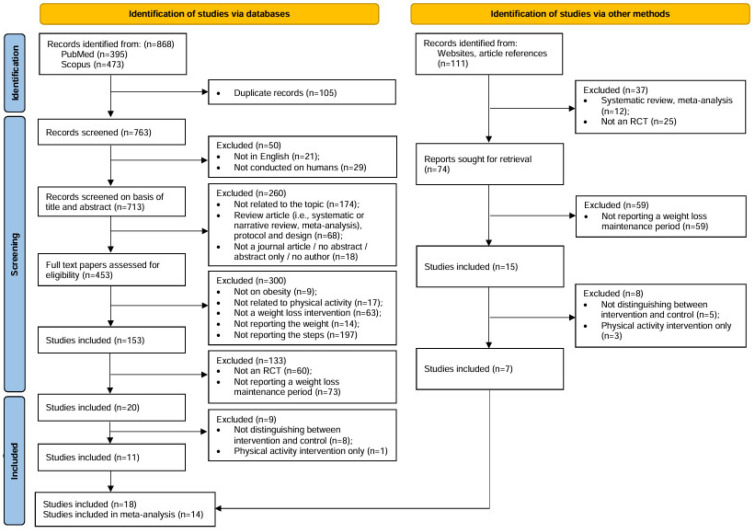
Flowchart summarizing the study selection procedure.

At the end of the selection process, 18 RCTs were available for the systematic review, and 14 of those underwent meta-analysis [57,58,59,60,61,62,63,64,65,66,67,68,69,70,71,72,73,74] (Figure 1). The first author, year of publication, country of conduction, study sample (stratified by gender), age, body mass index (BMI) at baseline, daily steps at baseline, type of intervention and total duration of the study are reported in Table 1. The Jadad scale checklist indicated that the RCTs (*n* = 18) had a moderate to high methodological quality (mean score 3.83 points). However, the assessment reflects selected domains of study design and does not represent a comprehensive evaluation of the overall risk of bias (Table 2).

**Table 1:** Studies included in the systematic review.

First Author and Year	Country	Age and Gender (M/F)	BMI at Baseline	Steps at Baseline	Type and Duration of Intervention
De Greef et al., 2010 [57]	Belgium/USA	41 (28/13) I: 61.3 ± 6.3 years C: 61.3 ± 6.9 years	I: 29.0 ± 4.2 kg/m^2^ C: 31.5 ± 5.0 kg/m^2^	I: 7099 ± 4208 steps C: 5214 ± 3352 steps	CBT/52 weeks
Nakade et al., 2012 [58]	Japan	235 (116/119) M: I: 53.6 ± 6.7 years; C: 53.7 ± 6.3 years F: I: 55.1 ± 6.4 years; C: 54.2 ± 6.2 years	M: I: 29.8 ± 2.3 kg/m^2^; C: 30.5 ± 3.7 kg/m^2^ F: I: 30.9 ± 3.0 kg/m^2^; C: 31.1 ± 3.1 kg/m^2^	M: I: 7058 ± 2885 steps; C: 8337 ± 3651 steps F: I: 8122 ± 2928 steps; C: 7984 ± 3376 steps	BT/24 months
Fuller et al., 2014 [59]	Australia/Germany/United Kingdom	772 (104/668) CP: 46.5 (13.5) years SC: 48.2 (12.2) years	CP: 31.5 (2.6) kg/m^2^ SC: 31.3 (2.6) kg/m^2^	CP: 7068 (3364) steps SC: 7194 (3220) steps	BT/24 months
Fogelholm et al., 1999 [60]	Finland	85 (0/85) 29–46 years old	34.0 kg/m^2^	W1: 7973 (678) steps W2: 7383 (544) steps C: 6882 (705) steps	BT/52 weeks
Madjd et al., 2020 [61]	Iran United Kingdom	113 (0/113) CBT: 31 (6.2) years C: 31 (6.3) years	CBT: 31.19 (1.80) kg/m^2^ C: 31.24 (1.66) kg/m^2^	CBT: 5757 ± 551 steps C: 5897 ± 537 steps	CBT/30 weeks
Young et al., 2017 [62]	Australia	92 (92/0) Only WL: 49.0 (10.4) years WL + WLM: 49.5 (9.9) years	Only WL: 30.6 (3.4) kg/m^2^ WL + WLM: 30.8 (3.3) kg/m^2^	Only WL: 9276 (3162) steps WL + WLM: 7962 (2735) steps	BT/27 months
Morgan et al., 2013 [63]	Australia	159 (159/0) Online: 46.5 (11.1) years Resources: 48.0 (10.8) years Control: 48.0 (11.2) years	Online: 32.8 (3.4) kg/m^2^ Resources: 32.4 (3.3) kg/m^2^ Control: 33.1 (3.9) kg/m^2^	Online: 7449 (3145) steps Resources: 6461 (2715) steps Control: 6857 (2801) steps	BT/6 months
Ismail et al., 2019 [64]	United Kingdom	1742 (1490/252) 69.75 (4.11) years	Gr I: 28.17 (4.11) kg/m^2^ Ind. I: 28.31 (4.28) kg/m^2^ UC: 28.37 (4.59) kg/m^2^	Gr I: 6692.78 (2702.08) steps Ind. I: 6820.87 (2747.26) steps UC: 6781.18 (2708.37) steps	CBT/24 months
Wyke et al., 2019 [65]	United Kingdom Norway Portugal Netherland	1113 (1113/0) I: 45.9 (9.0) years C: 45.6 (8.7) years	I: 33.1 (4.6) kg/m^2^ C: 33.4 (4.7) kg/m^2^	I: 8438 (3211) steps C: 8306 (3146) steps	CBT/12 months
Van Dyck et al., 2013 [66]	Belgium USA	92 (69%/31%) 62 ± 9 years	I: 30.24 ± 2.62 kg/m^2^ C: 29.74 ± 2.95 kg/m^2^	I: 4959 ± 2414 steps C: 5139 ± 2933 steps	CBT/12 months
Nakata et al., 2019 [67]	Japan	95 (36/59) SH: 57.0 (5.7) years WS: 54.7 (6.6) years	SH: 28.2 (2.4) kg/m^2^ WS: 28.4 (3.1) kg/m^2^	SH: 6167 (2409) steps WS: 6246 (2689) steps	BT/27 months
Nakata et al., 2011 [68] Nakata et al., 2014 [69]	Japan	188 (43/145) EO: 51.7 (6.8) years GB: 50.7 (6.7) years C: 51.6 (6.2) years	EO: 29.2 (3.8) kg/m^2^ GB: 29.0 (3.0) kg/m^2^ C: 28.6 (2.8) kg/m^2^	EO: 6198 (2740) steps GB: 6435 (3016) steps C: 5677 (2565) steps	BT/30 months
Anderson et al., 2021 [74]	United Kingdom	560 (0/560) I: 58.8 (5.2) years C: 59.5 (5.7) years	I: 31.0 (4.7) kg/m^2^ C: 31.3 (4.3) kg/m^2^	I: 9723 (3677) steps C: 9182 (3404) steps	BT/12 months
Petrella et al., 2017 [70]	United Kingdom	80 (80/0) I: 49.1 (9.1) years C: 48.4 (9.1) years	I: 36.0 (5.9) kg/m^2^ C: 37.1 (6.1) kg/m^2^	I: 6859.6 (3253.8) steps C: 6483.8 (3407.7) steps	BT/12 months
James et al., 2015 [71]	Australia	80 I: 56.2 (12.6) years C: 58.1 (11.2) years	NR	NR	BT/20 weeks
Morgan et al., 2011 [72]	Australia	53 (53/0) I: 40.9 (6.7) years C: 40.3 (7.5) years	I: 33.3 (3.7) kg/m^2^ C: 33.1 (4.1) kg/m^2^	I: 8521 (2745) steps C: 8028 (2559) steps	BT/6 months
Bertz et al., 2012 [73]	Sweden	68 (0/68) DEG: 33.9 ± 4.5 years C: 32.2 ± 4.6 years	DEG: 29.9 ± 2.2 kg/m^2^ C: 30.2 ± 3.4 kg/m^2^	DEG: 9486 ± 3342 steps C: 8361 ± 3742 steps	BT/12 months

Abbreviations: M: male; F: female; BMI: body mass index; WL: weight loss; WLM: weight loss maintenance; BT: behavioral therapy; CBT: cognitive behavioral therapy; I: intervention group; C: control group; CP: commercial program; SC: standard care; WM: weight maintenance; W1: walking program 1; W2: walking program 2; Gr I: group intervention; Ind. I: individual intervention; UC: usual care; SH: self-help; WS: web-support; EO: education-only; GB: group support; DEG: diet and exercise group; NR: not reported.

**Table 2:** Assessment of risk of bias.

Author	De Greef et al. 2010 [57]	Nakade et al. 2012 [58]	Fuller et al. 2014 [59]	Fogelholm et al. 1999 [60]	Madjd et al. 2020 [61]	Young et al. 2017 [62]	Morgan et al. 2013 [63]	Ismail et al. 2019 [64]	Wyke et al. 2019 [65]
Risk of bias									
Was the method of randomization to groups appropriate?	+	+	+	+	+	+	+	+	+
Was the allocation sequence concealed from those assigning patients to groups?	+	-	+	-	+	+	+	+	+
Was the outcome measurement performed in the same manner with similar intensity in all groups being compared?	+	+	+	+	+	+	+	+	+
Were similarly trained individuals administering the intervention across groups?	+	+	-	+	+	+	+	+	+
Were all the withdrawals described?	NR	NR	+	NR	+	+	+	+	+
Were all originally randomized participants analyzed in the groups they were assigned to (i.e., an intention-to-treat analysis)?	+	-	+	-	+	+	+	+	+
Was clustering at the group level accounted for in the analyses?	-	-	-	-	-	-	-	+	+
Were the groups similar at baseline?	+	+	+	NR	+	+	+	+	+
Jadad Scale									
Randomization	2	2	2	2	2	2	2	2	2
Blinding	1	0	1	0	1	2	2	1	1
Account of all patients	0	0	1	0	1	1	1	1	1
Total score	3	2	4	2	4	5	5	4	4
**Author**	**Van Dyck et al. 2013** [66]	**Nakata et al. 2019** [67]	**Nakata et al. 2011** [68]	**Nakata et al. 2014** [69]	**Anderson et al. 2021** [74]	**Petrella et al. 2017** [70]	**James et al. 2015** [71]	**Morgan et al. 2011** [72]	**Bertz et al. 2012** [73]
Risk of bias									
Was the method of randomization to groups appropriate?	+	+	+	+	+	+	+	+	+
Was the allocation sequence concealed from those assigning patients to groups?	-	+	+	+	+	+	-	+	+
Was the outcome measurement performed in the same manner with similar intensity in all groups being compared?	+	+	+	+	+	+	+	+	+
Were similarly trained individuals administering the intervention across groups?	+	+	+	+	+	+	-	+	+
Were all the withdrawals described?	NR	+	+	+	+	+	+	+	+
Were all originally randomized participants analyzed in the groups they were assigned to (i.e., an intention-to-treat analysis)?	+	+	+	+	+	+	-	+	-
Was clustering at the group level accounted for in the analyses?	-	-	-	-	-	-	+	-	-
Were the groups similar at baseline?	+	+	+	+	+	+	+	+	+
Jadad Scale									
Randomization	2	2	2	2	2	2	2	2	2
Blinding	0	1	2	2	1	2	0	1	1
Account of all patients	0	1	1	1	1	1	1	1	1
Total score	2	4	5	5	4	5	3	4	4

Risk-of-bias reporting: Yes: +; No: -; Not reported: NR. Jadad scale reporting randomized controlled trials. It evaluates three different items: randomization (0–2), blinding (0–2), and an account of all patients (0–1); studies with scores > 3 were considered good quality.

### 3.1. Narrative Synthesis

A total of four studies were not included in the meta-analysis, and their findings are summarized narratively.

In 1999, Fogelholm and colleagues [60] conducted a one-year trial on premenopausal (29–46 years) women with obesity (mean BMI = 34 kg/m^2^). The study consisted of a 12-week weight loss phase (WL) and a 40-week weight maintenance phase (WM). During weight maintenance, participants were randomized and allocated into three arms: control (C) and two walking programs aiming to expend 1000 kcal and 2000 kcal per week, respectively. During the WL phase, the mean weight loss did not differ between the three groups (−13.5 ± 0.6 kg vs. −13.0 ± 0.7 kg vs. −12.6 ± 0.7 kg; *p* = 0.62), or in daily steps (6882 ± 705 vs. 7973 ± 678 vs. 7383 ± 544; *p* = 0.49). During the weight maintenance phase, a significant increasing trend in daily steps per day was noticed which differed significantly between groups (C: 6607 ± 567 steps, walking group 1: 8303 ± 685 steps walking group 2: 9760 ± 830 steps; *p* < 0.001), but no difference in WL was found between the three groups (1.7 ± 0.8 kg vs. −0.7 ± 1.0 kg vs. 0.2 ± 0.9 kg; *p* = 0.18).

In 2014, Fuller and colleagues [59] reported a secondary analysis for a multicenter RCT to promote WL, which consisted of two arms: a commercial program (CP) and standard care (SC) extending over 12 months with a 24-month follow-up. Initially, participants were enrolled with an average age of 46.5 ± 13.5 years in the CP group and 48.2 ± 12.2 years in the SC group, with a BMI of 31.5 ± 2.6 kg/m^2^ and 31.3 ± 2.6 kg/m^2^, respectively. At 12 months, completers in the CP demonstrated significantly higher WL at 12 months compared to SC (−6.65 ± 0.43 vs. −3.26 ± 0.33 kg) [75]. In the secondary analysis [59], a significant increase in daily steps from baseline to 12 months was reported for the CP (7068 ± 3364 to 8152 ± 4052 steps) compared to SC (7194 ± 3220 to 7936 ± 3831 steps). At the 24-month follow-up, daily steps did not differ between the two arms (7875 ± 3136 vs. 7624 ± 2952 steps) [59], and both groups gained weight, with the CP group gaining more (+2.0 kg) (*p* < 0.001) [59,76].

In 2015, James and colleagues [71] conducted a two-arm RCT consisting of an eight-week intervention and 12-week follow-up. The study enrolled 133 participants, with a mean age of 58.1 ± 11.2 years for the control group (C) and 56.2 ± 12.6 years for the intervention (I) group. Only 80 had a BMI greater than 25 kg/m^2^ and were included in the current systematic review. At the end of eight weeks the intervention group lost significantly more weight compared to control group (−2.1 (95% CI: −3.7 to −0.6) kg vs. 0.06 (95% CI: −0.6 to 0.8) kg; *p* < 0.001), while demonstrating a significant mean increase in daily steps of 960.1 (95% CI: −158.5 to 2078.8) compared to a decrease of −1370 (95% CI: −2722 to −18.1) in the control group. After 20 weeks, the intervention group maintained weight loss from baseline (−2.1 (95% CI: −3.5 to −0.7) kg) compared to −0.06 (95% CI: −1.0 to 0.8) kg for the C group. Concurrently, the intervention group maintained a mean increase in daily steps of 768.0 (95% CI: −751.7 to 2287.7) compared to a decrease of −1366 (95% CI: −2903 to 170.7) steps in the control group.

**Figure 2:**
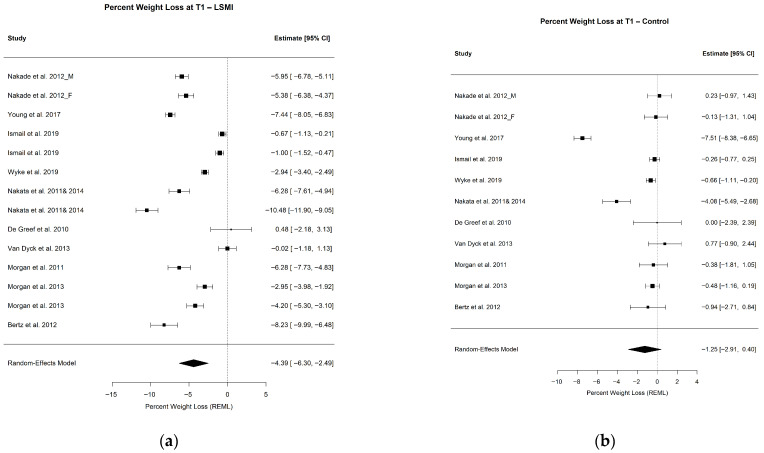
Forest plots for the pooled mean estimate of WL% at the end of the WL phase (T1) in (**a**) weight loss% in the intervention group and (**b**) weight loss% in the control group. Each square represent the effect estimate for an individual study with the size of the square proportional to the study weight. Horizantal lines indicate 95% confidence intervals (95% CIs). The diamond represents the pooled estimate with width correspondng to 95% CI. The vertical line denotes the line of no effect. The references of the studies included on the analysis at TI for LSMI: [57,58,62,63,64,65,66,68,69,72,73]; and for Control: [57,58,62,63,64,65,66,68,69,72,73].

In 2020, Madjd and colleagues [61] reported a two-arm, 24-week weight maintenance RCT. The study involved 113 women enrolled in either a cognitive behavioral therapy (CBT) group or a control group, with a BMI of 23–30 kg/m^2^, aged between 18 and 45 years, having lost at least 10% of their weight six months prior to the trial. At enrolment, the CBT group weighed 71.32 ± 5.52 kg, the control group weighed 72.14 ± 4.39 kg, and performed a mean of 5757 ± 551 steps per day and 5897 ± 537 steps per day, respectively. At the end of the weight maintenance phase, participants in the CBT group had a mean weight of 70.05 ± 4.63 kg compared to 72.76 ±4.30 kg in control group, while they walked around 7000 steps per day compared to 5000 steps per day in the control group (steps are shown in the original article, Figure 2) [61].

### 3.2. Meta-Analysis

A total of 14 studies were included in the meta-analysis with 31 arms (17 LSM arms and 14 control arms). The 14 studies included 3758 participants (1987 LSM arms and 1771 control arms) with a reported mean age of 52.71 ± 9.35 years (32.2–69.96 years). Sex was not discriminated across all studies; outcomes were either reported combined or the study included one sex, and only one study had separate interventions and control arms for males and females.

The meta-analysis was structured to first present WL outcomes to provide a clinical context, followed by an analysis of daily step counts as the primary behavioral outcome. Sensitivity analysis was then conducted to assess the robustness of the findings, and meta-regression analysis was subsequently performed as an exploratory analysis to examine the association between step counts and weight loss across studies.

#### 3.2.1. Pooled Estimate of WL%

**Figure 3:**
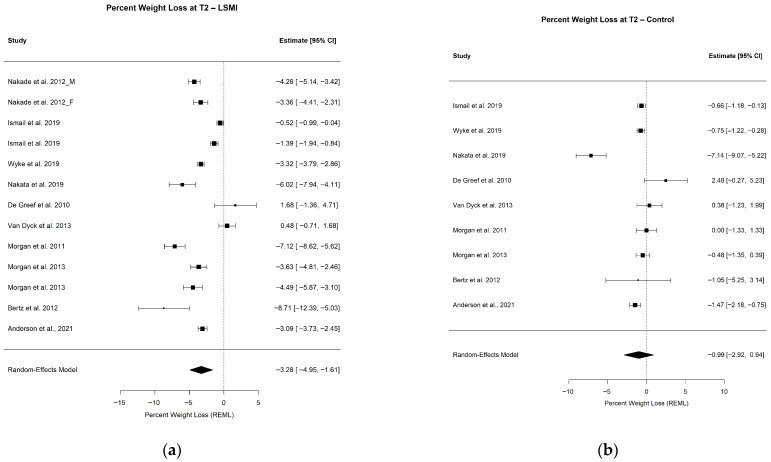
Forest plots for the pooled mean estimate of WL% at the end of WL maintenance phase (T2) in (**a**) weight loss% in the intervention group and (**b**) weight loss% in the control group. Each square represent the effect estimate for an individual study with the size of the square proportional to the study weight. Horizantal lines indicate 95% confidence intervals (95% CIs). The diamond represents the pooled estimate with width correspondng to 95% CI. The vertical line denotes the line of no effect. The references of the studies included on the analysis at T2 for LSMI: [57,58,63,64,65,66,67,72,73,74]; and for Control: [57,63,64,65,66,67,72,73,74].

The pooled mean estimate of the WL% in the LSM vs. control groups at each time point is presented in Table 3, as well as Figure 2a,b and Figure 3a,b. At the end of WL phase (T1), the participants in the LSM group lost over 4% (−4.39% (−6.30; −2.49); *p* < 0.001) of their initial weight at baseline (Figure 2a), while the control group lost close to 1% (−1.25% (−2.91; 0.40); *p* = 0.122), which did not deviate significantly from the line of no effect in the forest plot (Figure 2b). Comparing the pooled mean estimates of WL% at T1, WL in the group receiving LSM significantly exceeded the control by 3% (−3.14 (−5.39; −0.88); *p* = 0.006) (Table 3). The mean WL% at the end of maintenance phase (T2) decreased but remained significant for the LSM group (−3.28% (−4.95; −1.61); *p* = 0.001) while it remained of the same effect size of 1% for the control group (−0.99 (−2.92; 0.94); *p* = 0.272) (Table 3) (Figure 3a,b). The LSM group maintained over 2% (−2.29 (−4.52; −0.06); *p* = 0.044) higher WL% compared to the control group at T2. The WL% did not differ within group from T1 to T2 in both the LSM (1.11 (−1.18; 3.40); *p* = 0.342) and the control group (0.27 (−1.93; 2.46); *p* = 0.812) (Table 3). These findings provide the clinical context for the primary behavioral outcome, namely changes in the daily step counts.

#### 3.2.2. Pooled Estimate of Mean Daily Steps per Day

**Figure 4:**
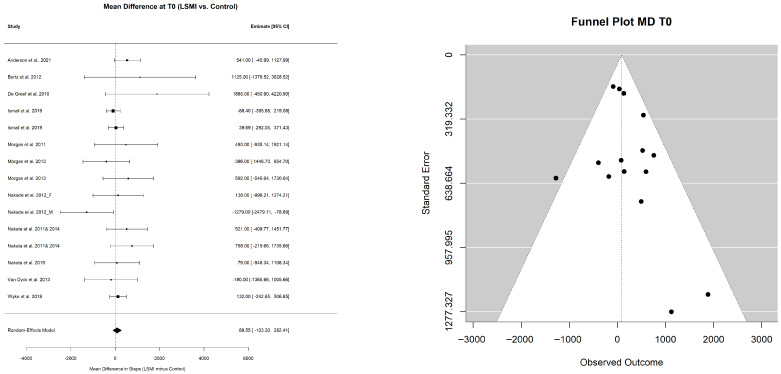
Forest plots and funnel plots for the pooled mean difference in daily steps between the LSM group and the control group at (T0). Each square represent the study specific mean difference (MD), with the size proportional to study weight. Horizantal lines indicate 95% confidence intervals (95% CIs). The diamond represents the pooled mean difference, with width corresponding to 95% CI. The vertical line indicates no difference between groups (MD = 0). The references of the studies included in the analysis: [57,58,63,64,65,66,67,68,69,72,73,74].

**Figure 5:**
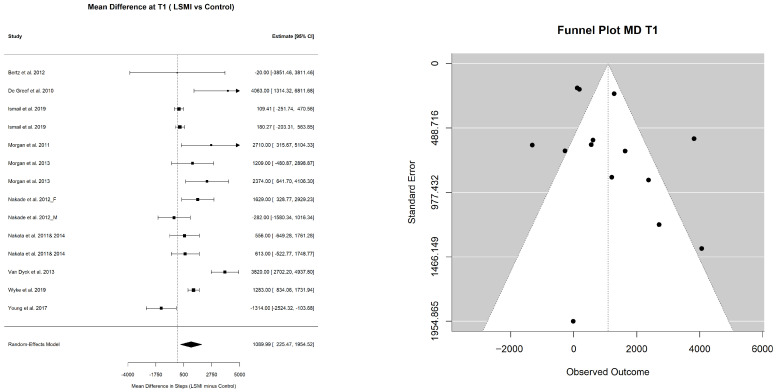
Forest and funnel plots for the pooled mean difference in daily steps between the LSM group and the control group at (T1). Each square represent the study specific mean difference (MD), with the size proportional to study weight. Horizantal lines indicate 95% confidence intervals (95% CIs). The diamond represents the pooled mean difference, with width corresponding to 95% CI. The vertical line indicates no difference between groups (MD = 0). The references of the studies included in the analysis at T1: [57,58,62,63,64,65,66,68,69,72,73].

**Figure 6:**
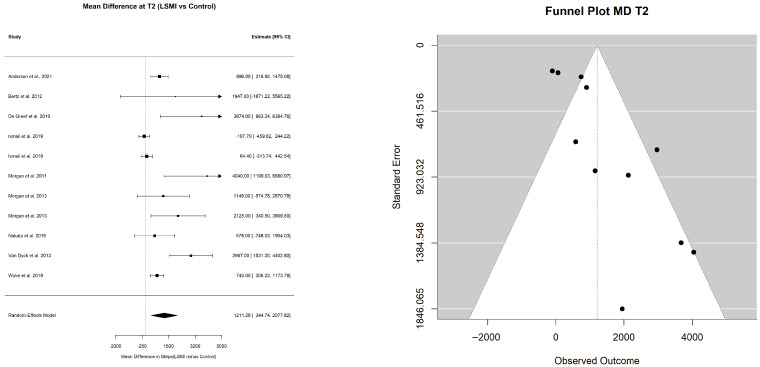
Forest and funnel plots for the pooled mean difference in steps between the LSM group and the control group at (T2). Each square represent the study specific mean difference (MD), with the size proportional to study weight. Horizantal lines indicate 95% confidence intervals (95% CIs). The diamond represents the pooled mean difference, with width corresponding to 95% CI. The vertical line indicates no difference between groups (MD = 0). The references of the studies included in the analysis: [57,58,63,64,65,66,67,72,73,74].

The pooled mean estimate for daily steps in the LSM and control groups, representing the primary behavioral outcome, in this study, at different time points, is presented in Table 4. The pooled mean daily steps at T0 did not differ between the LSM group (7253.21 (6576.97; 7929.44)) and control group (7129.71 (6328.65; 7930.76)) with a pooled MD of (89.6 (−103.3; 282.4); *p* = 0.336) (Table 5) (Figure 4). However, at the end of the intervention phase (T1), participants in the LSM group walked 8453.78 (7725.39; 9182.17) steps per day compared to 7486.13 (6313.37; 8658.88) steps per day in the control group (Table 4) with a mean pooled difference of 1090.00 ((225.5; 1954.5); *p* = 0.017) extra steps per day (Table 5) (Figure 5). At the end of the maintenance phase (T2) both groups decreased their steps, but the LSM maintained a higher step count per day (8241.28 (7302.30; 9180.26)) compared to control (6757.20 (5383.47; 8130.93)) (Table 4) with a pooled MD of 1211.28 ((344.74; 2077.82); *p* = 0.011) (Table 5) (Figure 6).

#### 3.2.3. Sensitivity Analysis

We conducted a sensitivity analysis using alternative correlation values (*r* = 0.6 to *r* = 0.9) for both outcomes. Sensitivity to the assumed correlations was observed, particularly for the steps outcome and for between groups in WL%, reflecting a non-linear variation in the pooled estimates across correlation values, likely driven by variance estimation and study weighting rather than inconsistency in the direction of intervention effect.

For WL%, the pooled estimates in the intervention group remained statistically significant at T1 and T2 across all assumption (*r* = 0.6, 0.7, 0.9, 0.95) with minor attenuation in effect size (*r* = 0.6: T1: WL% = −4.64, *p* < 0.001; T2: WL% = −3.60, *p* < 0.001; *r* = 0.95: T1: WL% = −4.39, *p* < 0.001; T2: WL% = −3.28, *p* < 0.001; *r* = 0.7: T1: WL% = −3.5, *p* < 0.001; T2: WL% = −2.56, *p* = 0.003). In contrast, between-group differences were significant only under *r* = 0.6 and the primary assumption (*r* = 0.95). Importantly, the primary assumption (*r* = 0.95) provided a more balanced contrast between intervention and comparator groups while maintaining statistical significance of the between-group difference, supporting its use as a reasonable assumption. This pattern was driven by variation in τ^2^, which influenced the total variance (Standard Error^2^ + τ^2^) and consequently the distribution of weights across studies [1/(Standard Error^2^ + τ^2^)]. Under alternative correlation assumption, higher τ^2^ values increased total variance, leading to more even distribution of weights across studies reducing between groups contrast (T1: difference attenuated form −3.14, *p* < 0.001 under 0.95 to −1.10, *p* = 0.417 under r = 0.9; T2: from −2.29, *p* = 0.044 to −0.10, *p* = 0.941) which support the use of the primary assumption (*r* = 0.95) consistent with prior literature [30].

As for steps, sensitivity analysis using alternative within-group correlation coefficients (*r* = 0.6, 0.7, 0.8 and 0.9) demonstrated that the direction of the effect consistently favored the intervention group at T1 and T2. However, the magnitude of the pooled MD varied across assumptions. Under the *r* = 0.6 and the primary assumption (*r* = 0.8), a statistically significant increase in daily steps was observed at T1 and T2 (T1:MD = 1303 and 1909 steps for *r* = 0.6 and 0.8, respectively; T2: MD = 1398 and 1211 steps at *r* = 0.6 and 0.8, respectively). In contrast, under alternative plausible values (*r* = 0.7 and *r* = 0.9), the pooled estimates were attenuated (T1: MD ≈ 474–549 steps; T2: MD ≈ 311–363 steps) and no longer statistically significant. Heterogeneity remained consistently high across all models with I^2^ values exceeding 80% for both weight loss% and MD daily steps, indicating considerable between-study variability independent of the assumed correlation coefficient.

Leave-one-out analysis for steps demonstrated that the pooled estimate was stable across correlation assumptions, with no single study altering the direction of statistical significance of the effect. However, the step MD estimates were significant under *r* = 0.6 and the primary assumption (*r* = 0.8). This pattern, as explained earlier, is driven by differences in variance estimation and, consequently, study weighing that is induced by the correlation assumption. However, the direction of the result consistently favored the intervention group across all analyses. Influence analysis supported these findings, showing no evidence of disproportionate influence by individual studies.

Leave-one-out analysis confirmed robustness of the LSM intervention effect on weight loss% with consistent significance across correlation assumptions. Usual care management estimates were more variable, and exclusion of one influential study reduced heterogeneity and yielded a significant estimate; however, the primary analysis remained non-significant, and the overall inference was unchanged. Influence analysis confirmed these findings.

#### 3.2.4. Association Between Step Counts and WL%

**Figure 7:**
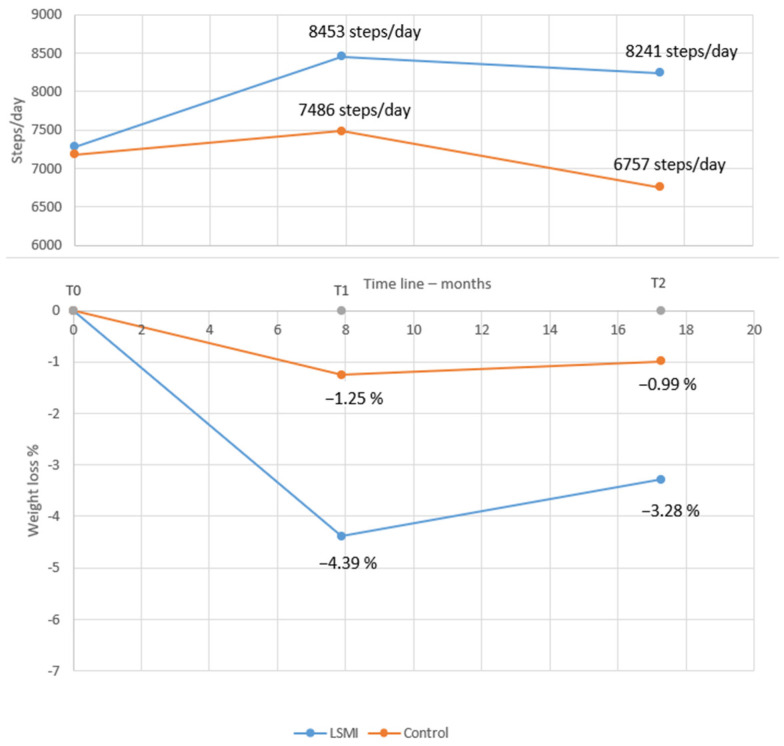
Pooled mean WL% by the weighted mean duration of intervention in months and the corresponding mean pooled steps per day.

The meta-regression analysis with daily step count per day, scaled to 1000 steps and used as a moderator at time T0, T1 and T2, showed a significant association between WL% at T2, and steps at T2 and T1. Such that, for every 1000 steps per day over baseline at T1, a 1.34% weight loss is achieved (−1.34; 95% CI (−2.54; −0.14); *p* = 0.033) and at T2, a 1.10% (1.10%; 95% CI (−1.96; −0.24); *p* = 0.017) WL is maintained in the LSM group from baseline (Figure 7).

#### 3.2.5. Risk of Reporting Bias

The potential risk of reporting bias was assessed by the funnel plots and the Egger test. Evidence for asymmetry of the funnel plots was suggested only for the pooled estimate of mean daily steps at T1 (*p* = 0.017) and pooled MD between LSM and control groups at T2 (*p* = 0.003), suggesting potential small study effect. Accordingly, a sensitivity analysis using fixed effects modeling for inverse variance meta-analysis for WL% was conducted and reflected the robustness of the random effect model with Hartung–Knapp correction for the overall analysis. The pooled mean estimates of the WL% were of the same size and direction in both the LSM and control groups. However, the pooled effect size of both the LSM and control groups was statistically significant in the fixed effects modeling assumption (Table S1), while only the LSM group maintained the significance of the intervention effect under a more conservative modeling (random effects with Hartung–Knapp correction) (Table 3). Suggesting that small studies may have influenced the magnitude but not the direction of the observed effects.

Regarding the pooled mean daily steps in the fixed effects model, large studies contributed the majority of the statistical weight, resulting in a pooled estimate reflecting larger studies (Table S2). Under the fixed effects model, the intervention group exhibited a modestly higher mean pooled step at T1 and not at T2 (Table S2), a pattern that reflects consistency with the random effect model and supports coherence of the results across different modeling assumptions. The sensitivity analysis for the estimate of pooled mean differences in step counts per day reflected consistency of findings across random-effects models with Hartung–Knapp correction and fixed effects inverse variance models (Table S3). The direction of the pooled mean difference, in steps per day, was consistently positive at the two time points and statistically significant, favoring intervention. Although the magnitude of the difference was smaller under fixed effects, this reflects merely a difference in weighting and heterogeneity handling between the models. These sensitivity analyses for mean WL% and daily step counts support the robustness of the results while accounting for the effect of between-study heterogeneity under random effect with Hartung–Knapp correction versus fixed effects modeling [31,35].

## 4. Discussion

This systematic review attempted to address three main questions of important clinical relevance and implications. Namely, the first is to quantify the levels of daily steps achieved under LSM interventions compared to the usual care management. The second is to examine when, across weight loss and maintenance phases, changes in step counts occur. The third is to explore how the timing of these changes is associated with weight management outcomes.

### 4.1. The Main Findings of the Systematic Review

This systematic review and meta-analysis revealed three main findings. First of all, on a general scale, it highlights the effectiveness of LSM programs for weight management of people affected by overweight or obesity with or without morbidities. In fact, our results showed that over a period of WL phase with mean duration of nearly eight months (range = 3–12 months), these programs appear to determine a reduction of more than 4% of body weight from baseline, and our finding is in line with others as reported in recently published systematic reviews that took into consideration LSM programs based on behavioral interventions for WL in patients with overweight or obesity that reported WL of a similar magnitude (≈5%) [77].

Following the WL phase, LSM programs appear to still determine a significant WL maintenance (range = 3–24 months) of the magnitude of 3.5% after a total mean duration of more than a 1.5-year follow-up from baseline (range = 6–30 months). Despite the fact that such a magnitude of WL maintenance may appear moderate, it can still be considered meaningful by some on the National Weight Control Registry (NWCR). For instance, the NWCR in Portugal considers a significant WL maintenance when ≥3% [78,79]. Accordingly, LSM programs should be, in any case, delivered as a first step and cornerstone intervention for overweight or obesity management, especially in those patients who have not previously undergone such interventions in a well-delivered way.

Second, in the LSM arm, an increase in the mean daily steps was observed during the WL phase, reaching a mean of nearly 8500 steps/day, and this level of daily steps was conserved during the weight maintenance phase; on the other hand, no significant changes were observed in the control arm at any time of follow-up with respect to baseline. Our finding is in line with previous published systematic reviews and meta-analyses on RCTs on the impact of LSM intervention based on behavioral strategies that clearly demonstrated that these programs increase the level of physical activity of patients as expressed in total duration (minutes per week) [80]. Moreover, our systematic review extends this evidence by suggesting that maintaining higher levels of daily steps during the weight maintenance phase may be associated with better long-term weight loss maintenance. Specifically, the mean daily steps achieved at the end of the WL phase and sustained during the maintenance phase were both associated with WL% maintenance at approximately 1.5 years of follow-up. However, this association should be interpreted cautiously, as it reflects study-level findings from meta-analysis rather than a prospectively validated threshold or a definitive clinical target.

Third, and of particular interest here underlining an important point that emerges from our results, is that the increase in the mean steps during the WL phase was not associated with the WL magnitude achieved during the WL phase, as we speculate that the increase in the level of physical activity during LSM programs may not help directly in losing weight during the WL phase, as apparently it is much more related to calorie restriction rather than to an active lifestyle (i.e., daily steps), but the same mean daily steps revealed to associate with maintenance of the WL achieved on a longer term. This finding highlights the important role of an active lifestyle during the maintenance phase rather than during the WL phase, as up until now, the impact of physical activity on WL and WL maintenance has not been consistently clarified [81].

### 4.2. The Clinical Implication

These findings are of clinical relevance and may inform discussions with treatment-seeking patients with overweight or obesity. The effectiveness of the LSM program in achieving a significant WL and maintenance in the long term should be highlighted. The current findings suggest that progressively increasing daily step counts from baseline through the WL phase—reaching around 8500 steps/day—and maintaining this level during the weight maintenance phase may be associated with improved long-term WL% maintenance. However, this should not be interpreted as a definitive or prescriptive clinical target—such a strategy may be considered in LSM programs; however, its effectiveness should be interpreted with caution, as the estimated between-group difference in daily step counts was sensitive to the variance assumption, and the meta regression analysis was exploratory in nature and does not establish a causal relationship. Accordingly, the proposed threshold (8500 steps/day) should be considered as hypothesis-generating rather than prescriptive. Further studies using predefined thresholds or dose–response analysis are needed to establish clinically meaningful step targets.

### 4.3. Strengths and Limitations

Indeed, this review has several strengths. Firstly, adhering to the PRISMA guidelines (File S1) and registering in PROSPERO, an international registry of systematic review protocols, gives our systematic review a methodological robustness and high standard of reporting that lends weight to the validity of the findings and conclusions, supported by a solid meta-analysis [26,27]. Secondly, the studies included in this systematic review and meta-analysis were exclusively RCTs [82], and as such were extremely well designed, featuring both randomized samples and appropriate control groups [83]. Thirdly, our systematic review included studies that assessed the daily steps objectively (not self-reported), by means of instruments amply validated and acknowledged in both clinical and research settings [84].

That being said, the review does have certain limitations. In particular, the lack of a sufficient number of studies in children and adolescents, as well as the paucity of studies that evaluated the daily steps during other weight management approaches, such as medications or bariatric surgery, and finally, the lack of studies with follow-ups that exceed 1.5 years in adults. All this limits the generalizability and external validity [85] of our findings regarding the impact of daily steps and weight management outcomes in those who underwent only LSM programs, and cannot be extended to the entire population with overweight and obesity.

In addition, several methodological considerations should be taken into account when interpreting the findings for the current meta-analysis. First, substantial between-study heterogeneity was observed, reflecting variability in intervention characteristics, participant profiles, and study designs. Differences in assumptions used for imputing change-score variances may have further contributed to this variability. Although sensitivity analysis was conducted, the pooled estimates—particularly for step count outcomes—remained sensitive to this assumption, yet maintained the direction of effect.

Second, the potential impact of risk-of-bias across studies should be considered. While most studies demonstrated appropriate randomization procedures and consistent outcome measurement, limitations in allocation concealment, incomplete reporting of withdrawals, lack of consistent intention-to-treat analysis and failure to account for clustering may have introduced selection, attrition and unit of analysis bias, affecting the precision of the pooled estimates.

Third, the potential impact of publication bias and small study effects cannot be excluded. Evidence of funnel plot asymmetry in some analyses suggests that smaller studies may have influenced the magnitude of the pooled estimates. However, sensitivity analysis (fixed effects vs. random effects with Hartung–Knapp, leave-one-out, and influence analysis) indicated that the overall direction of the finding remained consistent.

Overall, the certainty of evidence should be interpreted with caution. These methodological limitations, heterogeneity and sensitivity to analytical assumptions may influence the precision of the pooled estimates; therefore, the findings should be viewed as exploratory, particularly with respect to the magnitude of the observed effects.

Certainly, these aspects should be properly investigated in future studies, and as such, they represent new directions of needed research.

## 5. Conclusions

Our study supports the effectiveness of LSM programs in that they remain a valid approach for obesity management, achieving a modest but clinically meaningful WL (≈4–5%) and WL maintenance (≈3.5%), so they should be considered especially as a first-line intervention. Moreover, and according to our preliminary findings, during these programs, patients should be encouraged to adopt an active lifestyle with the aim to increase their average daily steps during the WL phase targeting approximately 8500 steps/day, and such a level should be maintained during the maintenance phase, an important strategy since it appears to be associated with maintaining significant WL in the long term. This needs to be openly and clearly discussed with patients before starting any LSM program due to the important clinical implications of this issue.

## Figures and Tables

**Table 3:** Mean pooled estimate of WL% in LSM and control groups at the end of WL phase (T1) and weight maintenance phase (T2).

Time Point	LSM Group						Control Group						Difference		
	Pooled Estimate % (95% CI)	*p* Value	K	I^2^	τ^2^	Egger Test *p* Value	Pooled Estimate% (95% CI)	*p* Value	K	I^2^	τ^2^	Egger Test *p* Value	Difference * (95% CI)	Z Value	*p* Value
T1	−4.39 (−6.30; −2.49)	<0.001	14	98.4%	10.32	0.784	−1.25 (−2.91; 0.40)	0.122	11	96.5%	5.75	0.600	−3.14 (−5.39; −0.88)	−2.72	0.006
T2	−3.28 (−4.95; −1.61)	0.001	13	97.2%	6.32	0.332	−0.99 (−2.92; 0.94)	0.272	9	96.1%	5.23	0.960	−2.29 (−4.52; −0.06)	−2.02	0.044
Within-group change (T2 → T1)	1.11 (−1.18; 3.40)	0.342					0.266 (−1.93; 2.46)	0.812							

* The difference reported is based on the Wald test.

**Table 4:** Mean pooled estimate of daily steps in LSM and control groups at T0 and at the end of WL phase (T1) and weight maintenance phase (T2).

Time Point	LSM Group						Control Group					
	Pooled Estimate % (95% CI)	*p* Value	K	I^2^	τ^2^	Egger Test *p* Value	Pooled Estimate% (95% CI)	*p* Value	K	I^2^	τ^2^	Egger Test *p* Value
T0	7279.69 (6555.65; 8003.73)	<0.0001	15	96.4%	1.55	0.551	7180.10 (6310.48; 8049.73)	<0.0001	12	95.7	1.66	0.621
T1	8453.78 (7725.39; 9182.17)	<0.0001	14	93.5%	1.29	0.017	7486.13 (6313.37; 8658.88)	<0.0001	11	95.9	2.60	0.512
T2	8241.28 (7302.30; 9180.26)	<0.0001	13	96.4%	1.95	0.061	6757.20 (5383.47; 8130.93)	<0.0001	9	97.2	2.75	0.937

**Table 5:** Meta-analysis summary mean difference in daily steps between LSM group vs. control group.

Time Point	Number of Studies	Pooled Mean Difference Steps (95% CI)	*p* Value	I^2^	τ^2^	Egger *p* Value
T0	15	89.6 (−103.3; 282.4)	0.3360	0.0%	0	0.254
T1	14	1090.0 (225.5; 1954.5)	0.0174	90.3%	1.65	0.239
T2	11	1211.3 (344.7; 2077.8)	0.0110	88.3%	1.02	0.003

## Data Availability

The original contributions presented in this systematic review are included in the article as results in the full text or in the appendices. Further inquiries can be directed to the corresponding author.

## Supplementary Materials

The following supporting information can be downloaded at: https://www.mdpi.com/article/10.3390/ijerph23040522/s1. Table S1: Fixed effect models for WL%; Table S2: Fixed effects models for pooled mean estimate of step count per day; Table S3: Fixed effects model for pooled mean difference in step counts of LSM vs. control; File S1: PRISMA_2020_checklist, Reference [86] is cited in the supplementary materials.
