# Supplementary material for: Daily Steps During Nutritional Lifestyle Modification Programs for Obesity Management: A Systematic Review and Meta-Analysis
Source: Int J Environ Res Public Health. 2026 Apr 17;23(4):522. doi: 10.3390/ijerph23040522 (PMC13116791; doi:10.3390/ijerph23040522)
Supplement: Supplementary file 1 [file ijerph-23-00522-s001.zip › ijerph-4170981-supplementary/ijerph-4170981-supplementary.pdf]

Sensitivity Analysis Using Fixed Effects Models

Table S1. Fixed effect models for WL%

	LSM group			Control group		
Time point	Mean	CI	P value	Mean	CI	P value
T1	-3.30337	(-3.513, -3.094)	<0.0001	-1.1345152	(-1.389, -0.88)	<0.0001
T2	-2.43974	(-2.663, -2.216)	<0.0001	-0.8410775	(-1.12, -0.562)	<0.0001

WL%= weight loss percentage LSM= lifestyle modification; CI= confidence interval; T1= time at the end of WL phase; T2= time at the end of WL maintenance phase.

Table S2. Fixed effects models for Pooled mean estimate of step count per day

Group	Time point	Mean	CI
LSM	T0	7,236.254	(7123.752, 7348.756)
LSM	T1	7,691.021	(7548.831, 7833.211)
LSM	T2	7,418.859	(7284.473, 7553.244)
Control	T0	7,488.962	(7348.666, 7629.257)
Control	T1	7,486.547	(7314.887, 7658.207)
Control	T2	7,496.816	(7332.692, 7660.94)

WL%= weight loss percentage LSM= lifestyle modification; CI= confidence interval; T0= time at baseline; T1= time at the end of WL phase; T2= time at the end of WL maintenance phase.

Table S3. Fixed effects model for Pooled mean difference in step counts LSM vs. Control

Time point	Number of studies	Pooled mean difference steps	95 percent CI lower	95 percent CI upper	P value	I^2	Egger test p value
T0	15	89.6	-73.7	252.8	0.282	14.2%	0.205
T1	14	587.8	386.2	789.3	<0.0001	83.7%	<0.001
T2	11	399.8	201.4	598.2	<0.0001	78.0%	<0.001

LSM= lifestyle modification; CI= confidence interval; T0= time at baseline; T1= time at the end of WL phase; T2= time at the end of WL maintenance phase; I^2= heterogeneity.
